# Disrupted Cerebellar Connectivity With the Central Executive Network and the Default-Mode Network in Unmedicated Bipolar II Disorder

**DOI:** 10.3389/fpsyt.2018.00705

**Published:** 2018-12-18

**Authors:** Xiaomei Luo, Guanmao Chen, Yanbin Jia, JiaYing Gong, Shaojuan Qiu, Shuming Zhong, Lianping Zhao, Feng Chen, Shunkai Lai, Zhangzhang Qi, Li Huang, Ying Wang

**Affiliations:** ^1^Medical Imaging Center, First Affiliated Hospital of Jinan University, Guangzhou, China; ^2^Institute of Molecular and Functional Imaging, Jinan University, Guangzhou, China; ^3^Department of Psychiatry, First Affiliated Hospital of Jinan University, Guangzhou, China; ^4^The Sixth Affiliated Hospital of Sun Yat-sen University, Guangzhou, China; ^5^Department of Radiology, Gansu Provincial Hospital, Lanzhou, China

**Keywords:** bipolar disorder, functional magnetic resonance imaging, cerebellar-cerebral functional connectivity, default-mode network, central executive network

## Abstract

**Objective:** Bipolar disorder (BD) is a common psychiatric disease. Although structural and functional abnormalities of the cerebellum in BD patients have been reported by recent neuroimaging studies, the cerebellar-cerebral functional connectivity (FC) has not yet been examined. The present study aims to investigate the FC between the cerebellum and cerebrum, particularly the central executive network (CEN) and the default-mode network (DMN) in bipolar II disorder (BD II).

**Methods:** Ninety-four patients with unmedicated BD II depression and 100 healthy controls (HCs) underwent the resting-state functional magnetic resonance imaging. Seed-based connectivity analyses were performed using cerebellar seeds previously identified as being involved in the CEN (bilateral Crus Ia) and DMN (bilateral Crus Ib).

**Results:** Compared with HCs, BD II depression patients appeared decreased FC in the right Crus Ia-left dorsal lateral prefrontal cortex (dlPFC) and -left anterior cingulate cortex (ACC), the right Crus Ib-left medial prefrontal cortex (mPFC), -left middle temporal gyrus (MTG), and -left inferior temporal gyrus (ITG). No altered FC between the left Crus Ia or Crus Ib and the cerebral regions was found.

**Conclusions:** Patients with BD II depression showed disrupted FC between the cerebellum and the CEN (mainly in the left dlPFC and ACC) and DMN (mainly in the left mPFC and temporal lobe), suggesting the significant role of the cerebellum-CEN and -DMN connectivity in the pathogenesis of BD.

## Introduction

Bipolar disorder (BD) is a chronic, severe and fluctuating psychiatric disease and receives widespread attention due to its various clinical manifestation, complicated course, and difficulty in treatment. Though BD consists of recurring episodes of mania and depression, the most common manifestation is the depressive episode, which results in a misdiagnosis as a major depressive disorder (MDD), bringing about mistreatment, huge medical costs, and poor clinical outcome. Despite being a common and important psychiatric illness, the specific pathogenesis of BD remains unclear. The proposal that the cerebellum plays a significant role in the pathophysiological mechanisms of BD was raised these years (1). Some neuroimaging studies have demonstrated structural and functional abnormalities of the cerebellum in BD (2–4).

The cerebellum has traditionally been regarded as an important role in motor control, posture coordination, and linguistic processing. However, recently, some evidence from literatures highlight an association between the cerebellum and higher-order functions, such as non-motor cognition as well as emotion (5–7). Anatomically, the cerebellum of normal human has been divided into ten lobules named I–X, grouped as the anterior lobe (lobules I–V); posterior lobe (lobules VI–IX, including Crus I and Crus II and lobule VIIb); and the flocculonodular lobe (lobule X) (8, 9). And it has been shown to reciprocally connect with many brain regions, like the brainstem reticular nuclei, hypothalamus, periaqueductal gray matter, amygdala, prefrontal cortex, and anterior cingulate cortex (ACC) (10–12). These connections are hypothesized to be the neural substrates for the cerebellar-cerebral functional connectivity (FC). Functionally, several resting-state functional magnetic resonance imaging (rs-fMRI) data have demonstrated FC between the cerebellar subregions and cerebral networks. For instance, using voxel-based analysis, a study has reported strong FC between lobule Crus I, Crus II, and the frontoparietal network; between lobule IX, vermal VIIIb and the default-mode network (DMN); between lobules I–VI, VIII and the sensorimotor networks and so on in 228 normal humans (13). Performing independent component analysis in 15 normal humans, Habas et al. found that cerebellar Crus I and II mainly participated in the bilateral central executive network (CEN), lobule IX participated in the DMN, lobules V–VI participated in the sensorimotor networks, and lobule VI participated in salience network (14). Meanwhile, applying seed-based analysis in 40 normal humans, Krienen and Buckner have also found that subregions in cerebellum had FC with the CEN, DMN, and motor network (15).

The CEN was classically conceptualized as referring to many fields, including working memory, initiation, planning, inhibition, flexibility, and vigilance (16). And the DMN was suggested to subserve ongoing, or default functions of the brain (17). These two networks are recognized as 2 core neurocognitive networks (18), showing dysfunctional connectivity in neuropsychiatric disorders, such as Alzheimer's disease, MDD, and BD (19). Moreover, several rs-fMRI studies have displayed aberrant FC between the cerebellum and the CEN and DMN in MDD (20–22), and schizophrenia (23), suggesting that the cerebellum participated in the pathogenesis of psychiatric diseases. However, only one study paid close attention to the cerebellar FC in BD, which used cerebral seeds to probe the cerebral-cerebellar FC in BD with psychosis, and found cerebral-cerebellar dysconnectivity in selective networks, such as the somatomotor, ventral attention, salience, and frontoparietal networks (24).

BD has two main subtypes, bipolar I disorder (BD I), and bipolar II disorder (BD II), which present different affective states and personality characteristics. According to the Diagnostic and Statistical Manual of Mental Disorders, Fifth Edition (known as DSM-V), BD I have a manic episode while BD II have a hypomanic episode. Though patients with BD II have less severe intensity, several studies suggest that patients with BD II may have a more chronic course, higher frequency of depressive episodes and comorbidity, more suicidal behavior, and rapid cycling than patients with BD I (25–29). There are reports about the genetic (28, 30, 31), metabolic (32), and electrophysiological (25, 33, 34) differences between BD II and BD I. And neuroimaging studies have shown the differences in structure (29, 35–39), and task-based function (40–42) between BD II and BD I. However, most neuroimaging studies, especially rs-fMRI studies, recruited heterogeneous samples of varying BD subtypes that included BD I, BD II. To date, the neurobiology of BD II has been poorly investigated. No prior study has researched the FC between cerebellum and cerebrum in BD II.

In the present study, we collected rs-fMRI data from patients with unmedicated BD II depression and healthy controls (HCs) at relatively large sample scale. To investigate the intrinsic cerebellar-cerebral FC in the CEN and DMN in BD II depression patients and HCs, seed-based correlation analyses were conducted. Cerebellar seeds were used to identify the CEN, and DMN, which were demonstrated to have a cerebral-cerebellar connection by previous studies (7, 15, 21, 22). We hypothesized that BD II depression patients would have aberrant FC in the cerebellum -CEN and -DMN.

## Methods and Materials

### Subjects

A total of 200 individuals ranging in age from 18 to 55 years participated in this study, including 97 currently depressed adults diagnosed with BD II and 103 HCs. The patients were recruited from the psychiatry department, First Affiliated Hospital of Jinan University, Guangzhou, China. All patients met DSM-V criteria for BD II according to the diagnostic assessment by the Structured Clinical Interview for DSM-V Patient Edition (SCID-P). The clinical state was assessed by using the 24-item Hamilton Depression Rating Scale (HDRS) and the Young Mania Rating Scale (YMRS) during the 3-day period prior to the imaging session. To be eligible for the study, all patients should have a total HDRS-24 score of > 21 and total YMRS score of < 7. At the time of testing, all patients were either medication-naïve or were not medicated for at least 6 months. The exclusion criteria included the presence of (a) any other Axis-I psychiatric disorders, (b) a history of electroconvulsive therapy, (c) a history of organic brain disorder, mental retardation, neurological disorders, (d) pregnancy, alcohol/substance abuse or dependence, or any presence of a concurrent and major physical illness, (e) any contraindication to MRI scanning. In addition, 103 right-handed HCs were recruited through local advertisements. To rule out the presence of a current or past history of any psychiatric illness, HCs were carefully screened through a diagnostic interview-the Structured Clinical Interview for DSM-V Non-patient Edition (SCID-NP). Further exclusion criteria for HCs were any history of a current or past significant medical or neurological illness, psychiatric illness in first-degree relatives.

This study was approved by the Ethics Committee of First Affiliated Hospital of Jinan University, Guangzhou, China, and signed a written informed consent for each participant after a full explanation of the study. Two senior clinical psychiatrists confirmed that all subjects had the ability to consent to participate in the examination.

### MRI Data Acquisition

Imaging was performed on a GE Discovery MR750 3.0 T System with an 8-channel phased array head coil. Subjects were scanned in a supine, head-first position with symmetrically placed cushions on both sides of the head to decrease motion. Before the scanning, each participant was repeatedly instructed to relax with their eyes closed without falling asleep; after the experiment, only participants who confirmed that they had not fallen asleep were included; otherwise, he/she was excluded.

The rs-fMRI data were acquired using gradient-echo echo-planar imaging sequence with the following parameters: time repetition (TR)/time echo (TE) = 2,000/25 ms, flip angle = 90°, voxel size = 3.75 × 3.75 × 3 mm3, field of view (FOV) = 240 × 240 mm, matrix = 64 × 64, slice thickness/gap = 3.0/1.0 mm, 35 axial slices covering the whole-brain, and 210 volumes acquired in 7 min. In addition, a three-dimensional brain volume imaging (3D-BRAVO) sequence covering the whole brain was used for structural data acquisition with: TR/TE = 8.2/3.2 ms, flip angle = 12°, bandwidth = 31.25 Hz, slice thickness/gap = 1.0/0 mm matrix = 256 × 256, FOV = 240 × 240 mm, NEX = 1, and acquisition time = 3 min 45 s. Routine MRI examination images were also collected for excluding anatomic abnormality. All participants were found by two experienced radiologists to confirm no brain structural abnormalities.

### Data Preprocessing

The preprocessing was carried out using Data Processing and Analysis of Brain Imaging (DPABI, http://rfmri.org/DPABI), which is based on Statistical Parametric Mapping (SPM12, http://www.fil.ion.ucl.ac.uk/spm/). For each subject, the first 10 images of the rs-fMRI dataset were excluded to ensure steady-state longitudinal magnetization. The remaining 200 images were first slice-time corrected and then were realigned to the first image for correcting for inter-TR head motion. This realignment correction provided a record of the head motion within the rs-fMRI scan. All subjects should have no more than 2 mm maximum displacement in any plane, 0.2 mm in mean frame-wise displacement (FD) (43), and 2° of angular motion. The individual T1 structural images were segmented (white matter, gray matter, and cerebrospinal fluid) using Segmentation toolbox. Then, the Diffeomorphic Anatomical Registration Through Exponentiated Lie algebra (DARTEL) toolbox was used to create a study specific template for the accurate normalization. Then, resting-state functional images were coregistered to the structural images and transformed into standard Montreal Neurological Institute (MNI) space, resliced to a voxel size of 3 × 3 × 3 mm3 resolution and smoothed using a 4 mm full width at half maximum (FWHM) Gaussian kernel. Furthermore, the data were removed linear trend and passed through band-pass filtered of 0.01–0.1 Hz. The Friston-24-parameter model of head motion (including the 6 head motion parameters, 6 head motion parameters one time point before, and the 12 corresponding squared items) (44) was chosen based on prior work (45). Several spurious covariates and their temporal derivatives were then regressed out from the time course of each voxel, including the signals of the brain global mean, white matter, and cerebrospinal fluid as well as the Friston-24 parameters of head motion.

### FC Analysis

**Figure 1:**
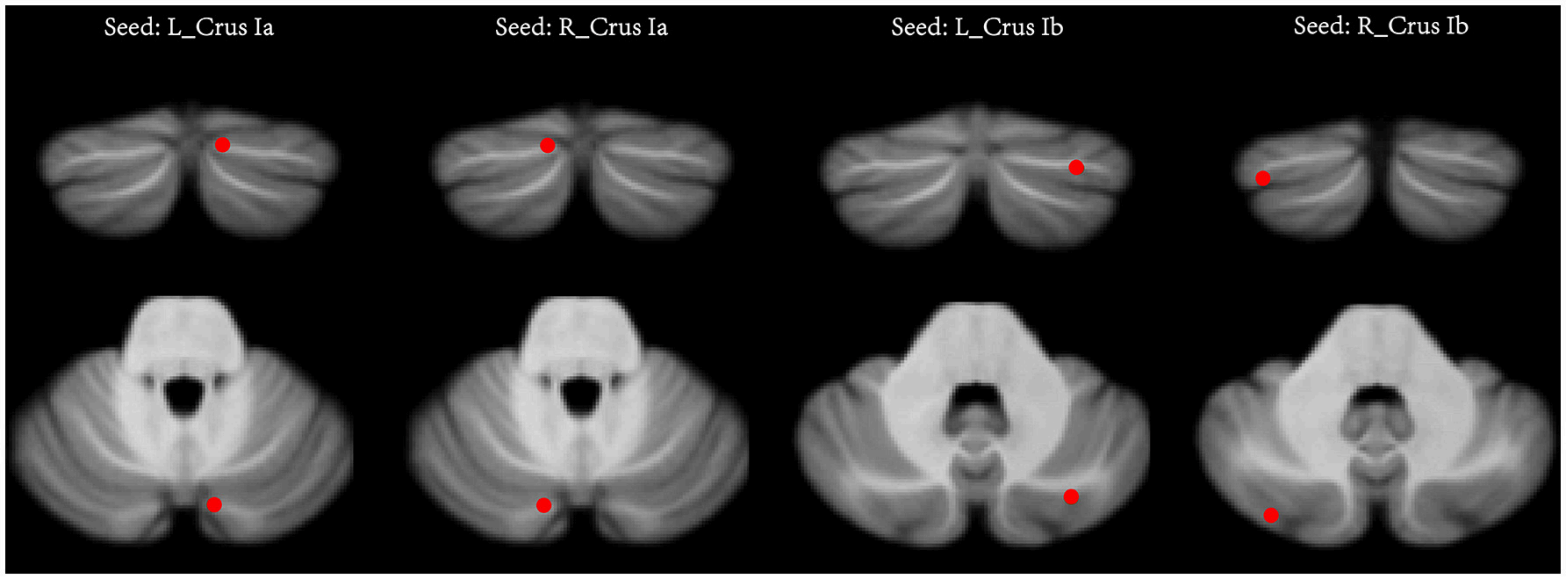
The seeds of the cerebellum. In each hemisphere, two seeds were defined, including the Crus Ia and Crus Ib. L (R), left (right) hemisphere.

Two previous seed-based rs-fMRI studies have reported that the left cerebellar Crus Ia (MNI coordinates: −12, −78, −28) and right cerebellar Crus Ia (MNI coordinates: 12, −78, −28) participated in the CEN, and the left cerebellar Crus Ib (MNI coordinates: −32, −76, −34) and right cerebellar Crus Ib (MNI coordinates: 34, −80, −36) participated in the DMN in major depressive disorder (21, 22). It was noticed that the bilateral Crus Ib involved in the DMN were asymmetric. These two seeds were defined by Krienen and Buckner in a study that used frontal seeds to investigate the FC between the cerebrum and cerebellum in normal humans (15). They found that the DMN (mPFC) was strongly connected with the bilateral Crus Ib. Thus, we chose these four seeds with a radius of 6 mm to examine the FC between the cerebellum and the CEN and the DMN (Figure 1). The seed point reference time series of each seed was extracted by averaging the time series of voxels of each ROI. For each subject, correlation maps were produced by computing the Pearson's correlation coefficients between the time series of the seeds and all the other brain voxels. To improve the normality, the obtained correlation maps were then converted to *z*-values maps using Fisher's *r*-*to*-*z* transformation. For all the subjects, four *z*-score maps which represented the intrinsic FC of the four cerebellar seeds were finally obtained.

### Statistical Analysis

The two final group of participants included in this study were 94 patients with BD II (mean age, SD, gender ration) and 100 HCs. Independent-sample *t*-tests and χ^2^ tests were used to compare the demographic data between the BD II and HCs groups with SPSS 17.0 software (SPSS, Chicago, IL, USA). All tests were two-tailed, and *p* < 0.05 were considered statistically significant.

The one-sample *t-*test was performed on z-score maps to demonstrate the within-group FC spatial distribution of each cerebellar seed for the BD patients and HCs. Then, the two-sample *t*-test was performed to assess the significant differences of the whole brain FC for each cerebellar seed between BD patients and HCs within the union mask of one-sample *t-*test results of both groups. Age, gender, education level, and the mean FD were included as nuisance covariates in the group comparisons. Statistical maps were thresholded using permutation test (PT) as implemented in Permutation Analysis of Linear Models (PALM) (46) and integrated into DPABI. The threshold-free cluster enhancement (TFCE) and voxel-wise correction (VOX) with PT were tested at two-tailed *p* < 0.05 for multiple comparisons. The number of permutations was set at 1,000.

Once statistically significant group differences were observed in any brain FC, we calculated the Pearson correlation coefficients between the clinical variables and FC values in BD II group. These clinical variables included onset age of illness, number of episodes, duration of illness, HDRS score, and YMRS score.

## Results

### Demographic Information

Table 1 shows the demographic and clinical information of all study participants. Three patients with BD II and three HCs were discarded from further analyses owing to excessive head movement during the image acquisition. Finally, the participants included 94 BD II patients and 100 HCs. The two groups have no significant differences in sex, age, years of education, and FD parameter.

**Table 1:** Demographic and Clinical Data in BD II patients and HCs.

**Variables**	**BD II**	**HCs**	***P*-Value**
No. of participants	94	100	
Gender (M: F)	51:43	45:55	0.198^**^
Age (years)	27.18 ± 9.15	28.32 ± 8.95	0.383^*^
Years of education	14.06 ± 2.79	14.72 ± 2.76	0.101^*^
Age at onset (years)	22.36 ± 8.94	NA	
No. of episodes	3.01 ± 1.45	NA	
Duration of illness (months)	47.11 ± 60.51	NA	
24-item HDRS score (points)	26.02 ± 6.80	NA	
YMRS score (points)	3.98 ± 5.27	NA	
FD values (mm)	0.08 ± 0.04	0.08 ± 0.04	0.497^*^

*Unless otherwise indicated, data are means ± standard deviation. BD, bipolar disorder; HCs, healthy controls; HDRS, Hamilton Depression Rating Scale; YMRS, Young Mania Rating Scale; FD, framewise displacement for in-scanner head motion; NA, not applicable*.

** *P-value for sex distribution obtained by χ^2^ test*.

* *P-values obtained by independent sample tests*.

### FC Analysis Within-Group

One-sample *t*-tests displayed the within-group FC patterns in the BD II and HCs group. Within the HCs group, the FC spatial distribution of the bilateral cerebellar Crus Ia is mainly located in the CEN, including the bilateral dlPFC, ACC, and superior parietal cortext. And the FC spatial distribution of the bilateral cerebellar Crus Ib is mainly located in the DMN, including the bilateral mPFC, posterior cingulate cortex, precuneus, inferior parietal lobule, middle temporal gyrus (MTG), and inferior temporal gyrus (ITG). Visual inspection indicated that FC patterns of the bilateral cerebellar Crus Ia and Crus Ib in the BD II group were similar to those of the HCs group.

### FC Analysis Between-Group

**Figure 2:**
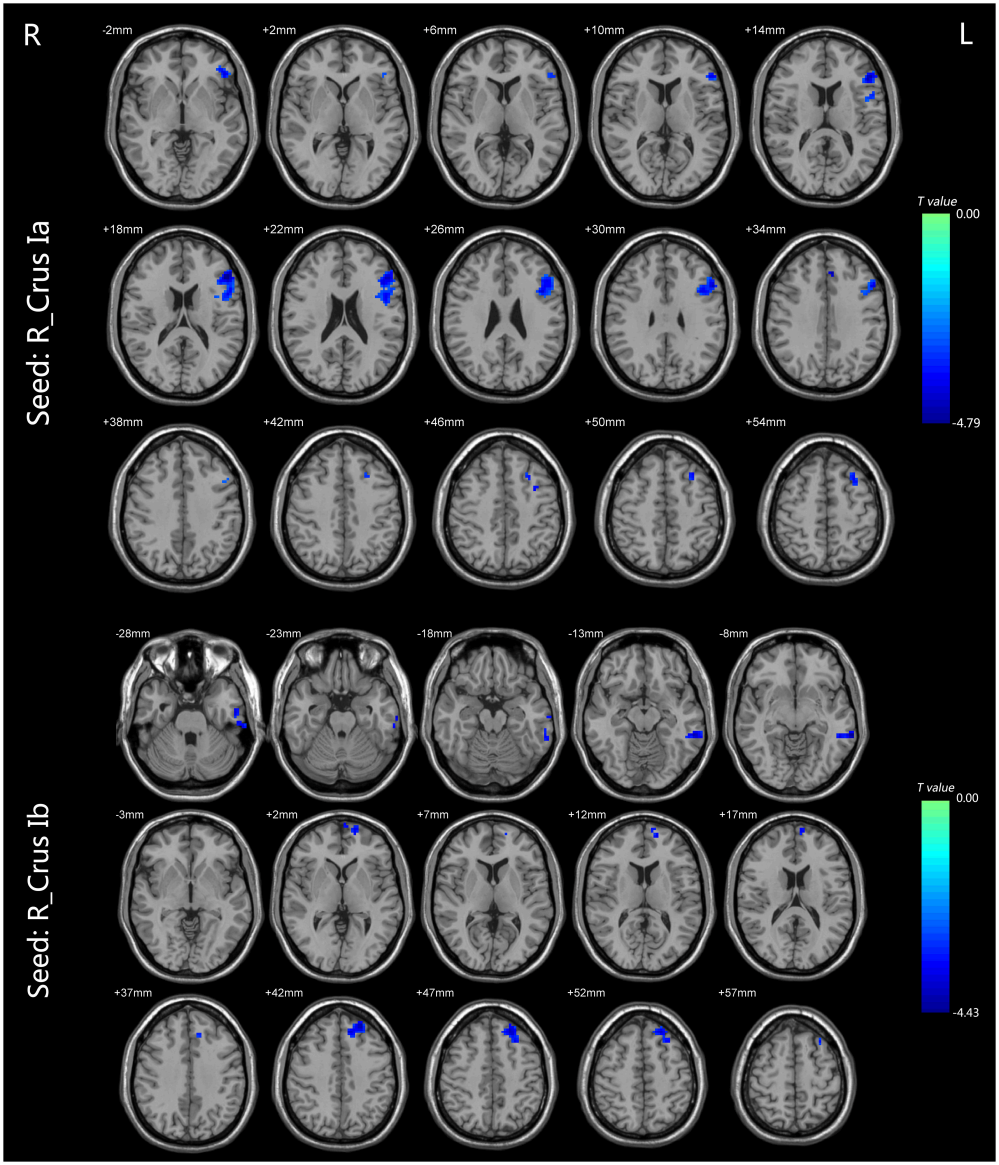
The FC differences between the BD patients and HCs (*p* < 0.05, TFCE corrected). For the CEN, the patients with BD II displayed decreased FC in the right Crus Ia-left dlPFC and left ACC compared with HCs. For the DMN, the patients with BD II displayed decreased FC in the right Crus Ib-left mPFC (vmPFC), left MTG, and left ITG (fusiform gyrus) compared with HCs. Shades of blue denoted decreased FC in the BD group compared with HCs group. The color bars indicate the *t*-values. FC, functional connectivity; BD, bipolar disorder; HCs, healthy controls; TFCE, the threshold-free cluster enhancement; CEN, central executive network; dlPFC, dorsal lateral frontal cortext; ACC, anterior cingulate cortext; DMN, default mode network; mPFC, medial prefrontal cortex; vmPFC, ventral medial prefrontal cortex; MTG, middle temporal gyrus; ITG, inferior temporal gyrus.

For the CEN, the patients with BD II displayed decreased FC in the right Crus Ia-left dlPFC, and -left ACC compared with HCs (Table 2, Figure 2). For the DMN, the patients with BD II displayed decreased FC in the right Crus Ib-left mPFC (ventral mPFC, vmPFC), -left MTG, and -left ITG (fusiform gyrus) compared with HCs (Table 2, Figure 2). There was no aberrant FC between the left Crus Ia or Crus Ib and the cerebral regions in BD II patients compared to the HCs. In addition, there was no increased FC in the BD II group relative to the HCs group. No significant correlations between the FC values in these regions and any clinical measure (including onset age of illness, number of episodes, duration of illness, HDRS, YMRS scores) in the BD II patients were found.

**Table 2:** Differences of cerebellar-cerebral FC between BD II patients and HCs.

**Cerebellar seeds**	**Significant regions**	***t* value**	**Cluster size (mm^3^)**	**side**	**MNI**			**Brodmann area**
					**x**	**y**	**z**	
BD < HCs								
R Crus Ia	dlPFC	−4.7902	810	L	−48	27	18	9, 46
	dlPFC	−3.8646	2160	L	−33	6	48	9
	ACC	−4.4720	81	L	−6	33	33	32
R Crus Ib	vmPFC	−3.5598	324	L	−15	57	12	10
	mPFC	−4.4338	1161	L	−24	42	45	8
	MTG	−4.3466	1242	L	−69	−39	−9	21
	ITG (fusiform gyrus)	−3.9390	594	L	−54	−15	−30	20

*FC, functional connectivity; BD, bipolar disorder; HCs, healthy controls; MNI, Montreal Neurological Institute; R, right; L, left; dlPFC, dorsal lateral prefrontal cortext; ACC, anterior cingulate cortext; vmPFC, ventral medial prefrontal cortex; mPFC, medial prefrontal cortex; MTG, middle temporal gyrus; ITG, inferior temporal gyrus*.

## Discussion

Using well-defined cerebellar seeds, we investigated the alteration of cerebellar-cerebral connectivity in BD II patients and found evidence of abnormalities in selective networks. Particularly, we found decreased cerebellar-cerebral FC in the CEN and DMN, particularly decreased Crus Ia-dlPFC, and ACC connectivity, and Crus Ib-mPFC, MTG, and ITG connectivity in unmedicated patients with bipolar II depression. This is the first study to report abnormal cerebellar connectivity with the CEN and DMN in patients with bipolar II depression.

The dlPFC and ACC are recognized as important regions within the CEN and are significant roles in maintaining cognition and emotion control (47–49). In this study, we found reduced FC within right Crus Ia-left dlPFC, and right Crus Ia-left ACC in patients with bipolar II depression, suggesting the impaired FC between the cerebellum and the CEN. Using seed-based connectivity analyses, a recent rs-fMRI found reduced cerebro-cerebellar FC in frontoparietal control networks in psychotic BD (24). Another rs-fMRI study reported decreased FC between the cerebellum and the dlPFC in patients with geriatric depression (21). Several studies demonstrated structural and functional abnormality of the dlPFC in BD. For example, several structural MRI studies reported reduced cortical thickness (50, 51) and gray matter volume (52) in the dlPFC in bipolar depression. Additionally, some rs-fMRI studies showed reduced FC between the dlPFC and mPFC (53), right temporal gyrus (54), left insula (55), and amygdala (56) in BD patients using seed-based approach or global brain connectivity method. Furthermore, task-based fMRI studies revealed that BD depressed patients showed decreased activation in the dlPFC and cerebellum during n-back tasks (57, 58). A previous PET study found that decreased dlPFC metabolism was associated with sustained attention deficits in depressed adults with BD (59). The ACC is a key region implicated in mood regulation (60) and might be a possible trait feature of BD (61, 62). An rs-fMRI study revealed decreased FC between the defined cingulo-opercular network (included the dorsal ACC attributed to cognition) and cerebellar network in BD (23). In addition, performing an emotion processing task, BD patients showed decreased activation in the ACC compared to HCs (63, 64). Therefore, disrupt connectivity of the cerebellar Crus Ia-dlPFC and -ACC may be associated with dysfunctional cognition and emotion control in BD II patients.

The DMN was suggested to subserve ongoing, or default functions of the brain such as self-referential mental activity, episodic memory retrieval, inner speech, mental images, emotions, and planning future events (17, 65). In this study, patients with BD II depression showed decreased FC in the cerebellum connected to the mPFC (mainly in the vmPFC). Using seed-based connectivity analyses, Alalade et al. reported that depression patients had reduced FC between the cerebellar seeds and the vmPFC (21). The mPFC is a core hub of the DMN in which abnormalities have been reported in BD (66). Structural MRI found decreased gray matter volume and density predominantly in the vmPFC in BD patients (67–69). And in rs-fMRI studies, Wang et al. found diminished functional connectivity strength (70) and voxel-mirrored homotopic connectivity (71) in the mPFC in non-medicine BD II patients. Using independent component analysis, Öngür et al. reported reduced DMN connectivity in the vmPFC in BD patients (72). Moreover, several studies demonstrated that ruminative self-focus was associated with the aberrant FC/activation in the mPFC in depression (73–76). A task-based fMRI study revealed that BD patients have decreased FC between the vmPFC and the head of the caudate, performing a self-reflection task (77). Taken together, our finding of the aberrant Crus Ib-mPFC connectivity may be involved in the abnormality of self-referential mental activity in BD patients.

The temporal gyrus is suggested to be in involved in several cognitive processing, including visual perception (ITG), language and semantic memory processing (MTG), sensory integration (78). In this study, the depressed BD II patients showed reduced FC between the right Crus Ib and left temporal lobe, including the MTG and ITG. A structural MRI study found reduced cortical thickness in the left MTG, ITG, and vmPFC in BD II patients (79). Two rs-fMRI studies demonstrated that the BD depression patients showed a reduction of functional connectivity strength in the left MTG (80) and reduction of the voxel-mirrored homotopic connectivity in the cerebellum and fusiform (81). Additionally, task-based fMRI studies found reduced activation in the MTG in BD II depression patients during an n-back task (82), and reduced activation in the MTG and ITG in BD I during an attentional task (83) and visuospatial processing task (84). Thus, these studies, united with our findings, suggest that the reduced FC within the Crus Ib-MTG and -ITG may be associated with disruption of cognition in BD.

What's more, unexpectedly, it's revealed that all the findings of reduced cerebellar-cerebral FC in patients appear between the right cerebellar seeds and the regions in the left cerebral hemisphere. There are pieces of evidence supporting abnormal hemisphere asymmetries in the major psychotic disorders, such as schizophrenia, MDD, and BD. Generally, a predominant left hemisphere disturbance in schizophrenia was reported by psychophysiological, neuropsychological, and neuroimaging studies (85–89). Electrophysiology, rs-fMRI and task-based fMRI studies showed a predominantly left-sided reduction of activity in the PFC in depression (90–94). And several studies found that patients with left hemisphere damage or lesions tended to appear depressive symptoms (95–97), while patients with right hemisphere damage or lesions tended to appear manic symptoms (97, 98). Two studies founded lower activation in the left hemisphere than in the right hemisphere in the BD depression while the opposite in the BD mania (99, 100), which is consistent with our finding of asymmetric left hemisphere disturbance in BD II depression. This phenomenon was reported to be associated with a high percentage of dopaminergic synapses in the left hemisphere, and the dopamine is essential in regulating motor activity, biological substrate of positive emotions and approaching behavior (99). Thus, the hemispheric asymmetry aberrance may be a significant biological substrate in BD.

There were several strengths in our study. First, we recruited a large number of participants of 94 BD II patients and 100 HCs, and the samples of non-medicine patients with BD II depression are homogeneous, while most previous rs-fMRI studies recruited heterogeneous samples of varying BD subtypes including BD I and BD II. Second, we noted permutation test with TFCE for multiple comparison correction in between-group contrast. A recent study demonstrated that the PT with TFCE, which is a strict multiple comparison correction strategy, was best able to reach the balance between family-wise error rate and test-retest reliability/replicability (101). However, there are also some potential limitations that should be taken into consideration. First, without a group of patients with BD in a euthymic episode, it is still not clear whether aberrant cerebellar-cerebral FC is specific to the depression episode of BD or shared by all episodes of the disease. Second, BD patients didn't participate in cognitive test scales, such as the Wisconsin Card Sorting Test, Digit Span Test, and Stroop Color Word Test. Further studies of the correlations between the impaired FC values and cognitive function are needed to support our speculations that the cerebellum-CEN and -DMN FC is involved in the cognition deficits in BD. Third, we didn't analyses the morphology characteristics of the BD patients, which should be considered and combined with our findings to provide more reliable imaging evidence in the pathogenesis of BD. Forth, further studies should be taken to investigate the differences of FC between BD II and BD I. Finally, we confirmed that the participants had not fallen asleep via their self-report, which could not ensure that each participant had not actually fallen asleep during the period of scanning.

## Conclusion

Patients with BD II depression showed disrupted FC between the cerebellum and the CEN (mainly in the left dlPFC and ACC) and DMN (mainly in the left mPFC, and temporal lobe), suggesting the significant role of the cerebellum-CEN and -DMN connectivity in the pathogenesis of BD.

## Author Contributions

XL, LH, and YW design the study; GC, YJ, JG, SQ, SZ, LZ, FC, SL, and ZQ contribute to data acquisition; GC contribute to data analysis; XL, GC, LH, and YW write the manuscript. All authors contributed to and have approved the final manuscript.

### Conflict of Interest Statement

The authors declare that the research was conducted in the absence of any commercial or financial relationships that could be construed as a potential conflict of interest.
